# Impact of medical professionals on Carbapenem-resistant *Pseudomonas aeruginosa*: moderating effect of workload based on the panel data in China

**DOI:** 10.1186/s12913-020-05535-5

**Published:** 2020-07-20

**Authors:** Meng Han, Xinping Zhang

**Affiliations:** School of Medicine and Health Management, Tongji Medical College, Huazhong University of Science and Technology, No.13. Hangkong Road, Wuhan, 430030 Hubei Province China

**Keywords:** Carbapenem-resistant *Pseudomonas aeruginosa*, Medical professionals, Workload, Moderating effect, Fixed effect model

## Abstract

**Background:**

Antimicrobial resistance (AMR), especially carbapenem-resistant *Pseudomonas aeruginosa* (CRPA), causes a serious increase in morbidity, mortality and costs. Medical professionals play an important role in curbing AMR. Previous studies overlooked the impact of workload on the relationship between medical professionals and AMR. This study aimed to explore the relationship between medical professionals and the CRPA rate as well as the moderating effect of medical professionals’ workload on this relationship.

**Methods:**

A provincial-level panel dataset from 2014 to 2017 was constructed. Medical professionals were measured by the numbers of physicians, registered nurses, pharmacists, and clinical microbiologists per 1000 population. Workload was measured by the number of daily physician visits. Fixed effect model and hierarchical regression analysis were performed to explore the moderating effect of workload on medical professionals and the CRPA rate.

**Results:**

The numbers of physicians, registered nurses, pharmacists and clinical technicians were significantly negative associated with the CRPA rate (coef. = − 0.889, − 0.775, − 1.176, and − 0.822; *P* = 0.003, 0.003, 0.011, and 0.007, respectively). Workload had a significant and positive moderating effect on physicians, registered nurses, pharmacists, clinical technicians and the CRPA rate (coef. = 1.270, 1.400, 2.210, and 1.634; *P* = 0.004, 0.001, 0.035, and 0.003, respectively).

**Conclusions:**

Increasing the number of medical professionals may help curb the CRPA rate. Measures aimed at reducing medical professionals’ workload should be implemented to further improve CRPA performance.

## Background

Many countries are facing the challenge of antimicrobial resistance (AMR). Approximately 25,000 people die from AMR per year in European countries [1]. A large number of newborn deaths caused by drug-resistant sepsis were also reported in India, Nigeria, Pakistan and the Democratic Republic of Congo [2]. AMR, particularly carbapenem-resistant *Pseudomonas aeruginosa* (CRPA), threatens global public health security [3]. As a main pathogen of healthcare-associated infections (HAIs), the treatment of *Pseudomonas aeruginosa* (*P. aeruginosa*) is often challenging due to its intrinsic non-susceptibility to many antimicrobials [4]. *P. aeruginosa* has become the third most common gram-negative cause of selected HAIs and it was estimated that approximately 51,000 HAIs resulting from *P. aeruginosa* occurred in the United States annually [5]. The increasing rate of CRPA worldwide limits its therapeutic options as well as aggravates the costs and the risk of mortality [6, 7]. According to the WHO, Carbapenems are considered as critically important antimicrobials (CIAs) and have become important solution to serious *P. aeruginosa* infections [5, 7].

Consistent with the WHO statement “antimicrobial resistance: No action today, No cure tomorrow” in 2011, establishing a multidisciplinary team of medical professionals has become a consensus in antimicrobial stewardship (AMS). The multidisciplinary team usually include an infectious disease physician, a clinical pharmacist and a clinical microbiologist, who are involved in the diagnosis, prescription, prudent use and evaluation of antimicrobials [8]. Registered nurses are often included in the team since they always serve as the most consistent providers of care at the bedside and are regard as one of the integral executors of AMS [9].

The relationship between medical professionals and AMR is a significant concern in health service research and health economics [10]. Prior studies mainly focused on the direct relationship between medical professionals and AMR. Liu et al. [10] found that increasing the number of clinical microbiologists could significantly reduce *Staphylococcus aureus* and coagulase-negative staphylococci rates by using provincial panel data in China. Another multicentre prospective cohort study found that pharmacist-led AMS programmes had a lower mortality rate (19.3% vs. 29.0%) and a lower multidrug-resistance rate (23.8% vs. 31.7%) compared with the control group [11].

Medical professionals’ workload is closely related to healthcare-related outcomes. Some previous studies have focused on the impact of workload on medical professionals. For instance, a systematic review pointed out that high workload harmed medical professionals’ well-being, such as job burnout and staff turnover [12]. Other studies have explored the relationship between medical professionals’ workload and AMR. A 3-year cohort study in Portugal showed that physicians’ heavy workload was associated with the poor quality of antimicrobial prescriptions (OR = 0.97; 95% CI: 0.94–1.00; *P* < 0.05) [13], which then could cause AMR. A similar result was also found in an observational study in Norway that general practitioners with heavier workload were more likely to prescribe antimicrobials for acute respiratory infections (OR = 1.64, 95% CI: 1.33–2.03, [14]).

Medical professionals and workload were regard as independent predictors of AMR in prior studies, but the potential interaction effects between medical professionals and their workload were always ignored. Therefore, an interesting question is whether workload can moderate the relationship between medical professionals and AMR. Furthermore, What is the action path of workload on this relationship? Since CRPA has arisen great concern, this study explored the relationship between medical professionals and the CRPA rate as well as the moderating effect of workload in this relationship using 2014–2017 provincial balanced panel data in China.

### Hypothesis

Hypothesis 1: The increased number of medical professionals is negatively associated with the CRPA rate. Specially, four different medical professionals play different critical roles in monitoring, guiding and rationally using antimicrobials and preventing CRPA as follows. Physicians prescribe necessary antimicrobial agents according to the patient’s symptoms and related guidelines. Nurses are directly involved in the administration, management and monitoring of antimicrobials, and they assess the patinet suitability for outpatient antimicrobial therapy, identify errors in a timely manner and provide appropriate education for patients [15]. Pharmacists play an integral role in educating physicians and patients on the appropriate use of antimicrobials, monitoring and auditing the outcomes of antimicrobial usage, and reviewing individual regimens for therapy optimisation [16]. Clinical microbiologists report on isolation and antimicrobial susceptibility tests that can provide evidence for the clinical antimicrobials selection [17]. As a poor practitioner-based referral system, it is common for medical professionals to receive approximately 100 patients daily in the outpatient departments in most tertiary hospitals, and consulting time for each patient is usually less than 3 min in China [18]. For these reasons, the time they spent on AMS and infection prevention and control is extremely limited. Increasing the number of medical professionals may help them better undertake these tasks.

Hypothesis 2: Workload moderates the association between medical professionals and the CRPA rate. Increasing the number of medical professionals can help improve hospitals’ service capacity, especially in terms of the monitoring, guidance, education, and the rational use of antimicrobials. However, medical professionals’ excessive workload may decrease the quality of medical care and nursing [19]. A retrospective study in Turkey found a significant association between increased nurse workload and multidrug-resistant organisms colonization or infection [20].

## Methods

### Variable measurement and data sources

A 4-year balanced panel dataset with 30 provinces in China from 2014 to 2017 were constructed. The main variables and data sources were as follows.

The numbers of physicians, registered nurses, pharmacists, and clinical microbiologists per 1000 population were selected as independent variables. The number of clinical microbiologists was estimated by technicians. Considering physicians, pharmacists, and clinical microbiologists are involved in the diagnosis, prescription, and prudent use of antimicrobials, and registered nurses are consistent executors, they are regarded as the core members of AMR stewardship [8, 9, 21]. The numbers of physicians, registered nurses, pharmacists, and clinical microbiologists were divided by 1000 population to adjust for the deviation caused by population differences.

The number of daily physician visits in hospitals was selected as the moderating variable. The increase in such daily visits indicates that medical professionals need to work longer or take fewer breaks to meet the medical demand, which has been regarded as one of the factors in measuring medical professionals’ workload [18]. For instance, Rodrigues et al. [13] used the number of daily physician visits as workload and found that workload influenced physicians’ antimicrobial prescriptions. Gjelstad et al. [14] explored general practitioners’ workload and their prescriptions by using the number of physician visits.

The number of tertiary hospitals was selected as the control variable. Patients generally tend to choose large general hospitals with high-quality medical resources because of the poor practitioner-based referral system in China. Tertiary hospitals are usually overcrowded and contain most of the cases of *P. aeruginosa* infections. CRPA rates, diagnostic proficiency, diagnostic availability and competence with infection control practices are hospital-specific in each province, which are especially influenced by the number of tertiary hospitals.

The rates of CRPA were extracted from the China Antimicrobial Resistance Surveillance System (CARSS) 2014–2017. The independent variables, moderating variable and control variable were extracted from the China Health and Family Planning Statistical Yearbooks 2015–2018 [22–25].

The summary statistics of the above variables are shown in Table 1. The number of registered nurses per 1000 population was the highest, followed by physicians, clinical microbiologists and pharmacists.

**Table 1:** Variable definitions and summary statistics

Variable	Definition	N	Mean	SD	Min	Max
CRPA	Rate of carbapenem-resistant *Pseudomonas aeruginosa*	120	21.445	6.514	8.7	36.4
PHY	The number of physicians per 1000 population	120	1.959	0.476	1.3	4.1
NUR	The number of registered nurses per 1000 population	120	2.524	0.525	1.65	4.8
PHA	The number of pharmacists per 1000 population	120	0.325	0.083	0.186	0.648
MCB	The number of clinical microbiologists per 1000 population	120	0.335	0.065	0.236	0.587
WORKL	The number of daily physician visits in hospitals	120	7.17	2.52	3.7	15.2
HOS	The number of tertiary hospitals	120	71.825	40.757	7	170

*N* Number; *SD* Standard deviation; *Min* Minimum; *Max* Maximum

### Statistical analysis

Hierarchical regression analysis was used to test the moderating effect. First, the following Eq. (1) was constructed to test the relationship between the number of medical professionals and the CRPA rate:

1 $$ \mathrm{Ln}\  CRPA\mathrm{i}\mathrm{t}=\upbeta\ 0+\upbeta\ 1\ \mathrm{Ln} STAFF\mathrm{i}\mathrm{t}+\upbeta\ 2\ \mathrm{Ln} HOS\mathrm{i}\mathrm{t}+\upmu\ \mathrm{i}+\upvarepsilon\ \mathrm{i}\mathrm{t}\kern0.5em , $$

Second, workload (Eq. (2)) and the interaction term between workload and the number of medical professionals (Eq. (3)) were gradually incorporated to test the moderating effect of workload in the relationship between the number of medical professionals and CRPA rate. The interaction terms in Eq. (3) were mean-centred to avoid multicollinearity effects. If the regression coefficients of interaction terms are statistically significant, then the moderating effect of workload is significant [26]:

2 $$ \mathrm{Ln}\  CRPA\mathrm{i}\mathrm{t}=\upalpha\ 0+\upalpha\ 1\ \mathrm{Ln}\  STAFF\mathrm{i}\mathrm{t}+\upalpha\ 2\ \mathrm{Ln}\  WORKL\mathrm{i}\mathrm{t}+\upalpha\ 3\ \mathrm{Ln}\  HOS\mathrm{i}\mathrm{t}+\upmu\ \mathrm{i}+\upvarepsilon\ \mathrm{i}\mathrm{t}, $$

$$ \mathrm{Ln}\  CRPA\mathrm{it}=\upgamma\ 0+\upgamma\ 1\mathrm{Ln}\  STAFF\ \mathrm{it}+\upgamma\ 2\ \mathrm{Ln}\  WORKL\mathrm{it}+\upgamma\ 3\ \mathrm{Ln}\  STAFF\ \mathrm{it} $$

3 $$ \ast \mathrm{Ln}\  WORKL\mathrm{i}\mathrm{t}+\upgamma\ 4\ \mathrm{Ln}\  HOS\ \mathrm{i}\mathrm{t}+\upmu\ \mathrm{i}+\upvarepsilon\ \mathrm{i}\mathrm{t}, $$

where i (i = 1, 2, 3..., 29, 30) indicates province, t (t = 1, 2, 3, 4) indicates year, μi is an unobservable regional effect, and εit is a random error term. STAFF indicates one type of medical professional, such as physicians, registered nurses, pharmacists, and clinical technicians. Each variable was taken in logarithmic form to eliminate the influence of heteroscedasticity.

The panel data model is a quantitative method for longitudinal data, which can increase the estimator precision by increasing observations and can obtain more dynamic information than single cross-sectional data [27]. The pooled ordinary least squares (OLS) model, fixed effect (FE) model and random effect (RE) model are often used to estimate panel data. Because OLS cannot control for the fixed effect of provinces, the estimation caused by endogeneity may be biased. Subsequently, the FE model or RE model can be employed for the estimation. The RE model is relatively more effective, but it requires that exogenous variables and individual effects are not correlated, while the FE model, although there is no requirement between exogenous variables and individual effects, consumes more degrees of freedom [28]. The Hausman test was used to determine the final model. As shown in Table 2, each of the tests rejected the null hypothesis, indicating that the FE model was more appropriate.

**Table 2:** Panel data model estimation

Variable	FE	RE	FE	RE	FE	RE	FE	RE
ln PHY	−0.888***	−0.706***						
ln NUR			−0.757***	− 0.833***				
ln PHA					−1.126***	−0.731***		
ln MCB							−0.785***	− 0.728***
ln WORKL	0.001	0.524***	0.181	0.537***	0.398	0.678***	0.296	0.486***
ln HOS	−0.418***	0.046	−0.316**	0.094	−0.443***	0.021	−0.433***	0.039
Constant	5.289***	2.285***	4.646***	2.359***	2.764**	0.790	3.345***	1.119**
Hausman test value	33.30***		24.72***		33.45***		34.28***	
P	0.000		0.0001		0.000		0.000	

*FE* Fixed effect (FE) model; *RE* Random effect (RE) model

*** *p* < 0.01, ** *p* < 0.05, and * *p* < 0.1

STATA (version 13.0, Stata Corp., College Station, Texas, USA) was used for the statistical analysis.

## Results

The 4-year balanced panel database included 120 records. Equations (1), (2) and (3) were tested in turn to explore the moderating effect of workload on the relationship between medical professionals and the CRPA rate.

### Correlation analyses amongthe variables

As shown in Table 3, workload was significantly and positively correlated with the CRPA rate (*r* = 0.363; *P* = 0.000). However, the associations between the numbers of physicians, registered nurses, pharmacists, and clinical technicians and the CRPA rate were not statistically significant (*r* = 0.120, 0.109, 0.095, and 0.136; *P* = 0.194, 0.236, 0.301, and 0.140, respectively).

**Table 3:** Correlation coefficients among medical professionals, CRPA rate and workload

Variable	1	2	3	4	5	6	7
1.ln PHY	1.000						
2.ln NUR	0.788***	1.000					
3.ln PHA	0.788***	0.794***	1.000				
4.ln MCB	0.731***	0.849***	0.766***	1.000			
5.ln WORKL	0.302***	0.388***	0.486***	0.225**	1.000		
6.ln HOS	0.109	0.128	0.086	−0.037	0.158*	1.000	
7. ln CRPA	0.120	0.109	0.095	0.136	0.363***	0.461***	1.000

*** *p* < 0.01, ** *p* < 0.05, and * *p* < 0.1

### Relationship between medical professionals and CRPA

As shown in Table 4, there were significant and negative associations between physicians, registered nurses, pharmacists, and clinical technicians and the CRPA rate (coef. = − 0.889, − 0.775, − 1.176, and − 0.822; *P* = 0.003, 0.003, 0.011, and 0.007, respectively). This finding indicated that the increase in medical professionals was associated with a lower CRPA rate. Thus, Hypothesis 1 was supported.

**Table 4:** Relationship between medical professionals and CRPA rate

Variable	Model (1)	Model (2)	Model (3)	Model (4)
ln PHY	−0.889***			
	(0.276)			
ln NUR		−0.775***		
		(0.240)		
ln PHA			−1.176**	
			(0.434)	
ln MCB				−0.822***
				(0.283)
ln HOS	−0.418***	−0.322**	− 0.465***	−0.445***
	(0.120)	(0.143)	(0.125)	(0.126)
Constant	5.302***	5.034***	3.560***	3.922***
	(0.354)	(0.403)	(0.959)	(0.791)
R-squared	0.431	0.457	0.404	0.412
F	66.77***	64.89***	50.88***	55.55***

*** *p* < 0.01, ** *p* < 0.05, and * *p* < 0.1; cluster-robust standard errors are in parentheses

### The moderating effect test of medical professionals workload

Models (5), (7), (9) and (11) presented significant and negative associations between the numbers of physicians, registered nurses, pharmacists, and clinical technicians and the CRPA rate after the addition of workload (coef. = − 0.888, − 0.757, − 1.125, and − 0.785; *P* = 0.005, 0.004, 0.017, and 0.008, respectively). Models (6), (8), (10) and (12) showed that the interaction terms of workload and medical professionals (ln WORKL* ln PHY, ln WORKL* ln NUR ln WORKL* ln PHA, ln WORKL* ln PHY, ln WORKL* ln MCB) were significantly and positively associated with the CRPA rate (coef. = 1.270, 1.400, 2.210, and 1.634; *P* = 0.004, 0.001, 0.035, and 0.003, respectively). The results indicated that workload moderated the impact of the number of medical professionals on the CRPA rate, and the increase in workload may lead to an increase in the CRPA rate. Therefore, Hypothesis 2 was supported (Table 5).

**Table 5:** Moderating effect of workload on the relationship between medical professionals and CRPA rate

Variable	Model (5)	Model (6)	Model (7)	Model (8)	Model (9)	Model (10)	Model (11)	Model (12)
ln PHY	−0.888**	−0.781**						
	(0.295)	(0.291)						
ln NUR			−0.757***	−0.683***				
			(0.241)	(0.221)				
ln PHA					−1.125**	−0.980**		
					(0.445)	(0.403)		
ln MCB							−0.785***	−0.739**
							(0.278)	(0.272)
ln WORKL	0.001	0.298	0.181	0.771*	0.398	0.702	0.296	0.702
	(0.503)	(0.526)	(0.469)	(0.434)	(0.602)	(0.640)	(0.546)	(0.570)
lnWORKL* ln STAFF		1.270***		1.400***		2.210**		1.634***
		(0.403)		(0.358)		(0.999)		(0.500)
ln HOS	−0.418***	−0.443***	−0.316**	−0.306**	−0.443***	−0.438***	− 0.433***	−0.426***
	(0.121)	(0.118)	(0.143)	(0.133)	(0.130)	(1.618)	(0.130)	(0.132)
Constant	5.289***	4.736***	4.646***	3.379	2.764*	2.250	3.345**	2.569*
	(1.228)	(1.237)	(1.167)	(1.057)	(1.608)	(1.618)	(1.463)	(1.481)
R-squared	0.431	0.452	0.458	0.500	0.410	0.439	0.415	0.447
F	55.98***	51.39***	49.46***	57.19***	39.76***	39.20***	43.98***	43.20***

*** *p* < 0.01, ** *p* < 0.05, and * *p* < 0.1; cluster-robust standard errors are in parentheses

ln STAFF refers to ln PHY in Models(5) and (6), ln NUR in Models(7) and (8), ln PHAin Models(9) and (10), and ln MCB in Models(11) and (12)

## Discussion

The following findings on the relationship between medical professionals and the CRPA rate as well as the moderating effect of medical professionals’ workload were worth discussing.

A 0.899% decrease in CRPA was associated with a 1% increase in the number of physicians, which was consistent with previous findings that reducing the misuse of antimicrobials by physicians can reduce the proportion of those colonized or infected with multidrug-resistant organisms [17]. Maeda M et al. [29] also found that the increased allocation of infectious disease physicians optimised antimicrobial therapy (11.4% vs. 18.5%, *P* = 0.012) and likely improved the clinical outcomes of patients with bloodstream infections. In addition, previous studies have found a significant and positive association between physician workload and antimicrobial prescriptions [14, 30]. Physicians with heavy workload found it time-consuming to discuss alternative therapeutic approaches with patients. The prescription of antimicrobials entailing an even broader spectrum of agents may thus be regarded as a time-saving strategy [14]. Therefore, the increased allocation of physicians allows them more time to ensure the appropriate prescription of antimicrobials and provide education for patients, which are conducive to contain AMR through the cooperation of physicians and patients.

There was an association between the increase in the number of registered nurses and the decrease in the CRPA rate (coef. = − 0.775; *P* = 0.003). A similar result was found in critical care populations that the risks of respiratory failure and reintubation increased when the nurse-patient ratio was adjusted from 1:3 into 1:1 or 1:2 [31]. Zhang et al. [32] found that the heavier the nursing workload was, the lower the adherence to hand hygiene. Sadule-Rios et al. [33] found that over half of nurses reported that the main barrier of low adherence to hand hygiene was their heavy workload. A shortage of nurses leads to the heavy workload [34]. Furthermore, heavy workload and lack of time are the main factors affecting adherence to hand hygiene, which can effectively and efficiently reduce the incidence of HAIs [33, 35]. Increasing the number of registered nurses allows them relatively sufficient time to improve adherence to regulations such as hand hygiene, ward disinfection and isolation systems, which can reduce the incidence of CRPA. The increase in registered nurses also allows them more time to monitor prescription decisions, follow up on missed doses and provide patients education [15].

This study also found that increasing the number of pharmacists may reduce the CRPA rate (coef. = − 1.176;*P* = 0.011). Similar to this, AMS programmes at Mount Sinai Hospital and the University Health Network showed that increasing the number of pharmacists to monitor and intervene in antimicrobial use led to approximately 15% drop in the consumption of antimicrobials in the ICU and a 5–17% increase in the susceptibility of isolates of *Pseudomonas* spp. to antimicrobials [36]. Zhang et al. [37] found a significant and negative association between rational use of carbapenems and the prevalence of CRPA.

AMS has become a platform for pharmacists because of the rising rate of AMR [38]. However, the implementation of clinical pharmacy is in the infancy in many developing countries [39]. There is a shortage of pharmacists to meet the growing demand of providing prescribing suggestions and promoting the rational use of antimicrobials in China [40, 41]. In particular, more than half of these pharmacists are concentrated in the more developed eastern regions of China [41]. Increasing the number of pharmacists can support prescribers much more fully in terms of optimising antimicrobial regimens and managing the entry of new antimicrobials into hospital formularies, including critical reviews of new agents and their place in treatments.

The increase in the number of clinical microbiologists was associated with the decrease in the CRPA rate (coef. = − 0.822;*P* = 0.007). Liu et al. [10] found a similar result that clinical microbiologists were regarded as a significant predictor of lower rates of *Staphylococcus aureus* and coagulase-negative staphylococci (coef. = − 0.191 and − 0.351; *p* = 0.070 and 0.004, respectively). Since clinical microbiologists are more familiar with the microbial characteristics of pathogens than other medical professionals, their recommendations for antimicrobial therapy are usually compelling and can ensure high degrees of compliance with treatment modifications. They can also help the clinic identify CRPA as soon as possible and provide appropriate targeted therapy based on antimicrobial susceptibility test results.

Workload had a moderating effect on the relationships among physicians, registered nurses, pharmacists, and clinical microbiologists and the CRPA rate (coef. = 1.270, 1.400, 2.210, and 1.634; *P* = 0.004, 0.001, 0.035, and 0.003, respectively), which meant that control workload would increase the impact of medical professionals on CRPA. It was confirmed that heavy workload was a main factor associated with poor hand hygiene adherence, which could lead to the spread of pathogens and the aggravation of AMR [32]. Excessive workload may cause medical professionals to neglect the implementation of monitoring, guiding, and the rational use of antimicrobials and infection control measures. Moreover, it may also hinder pharmaceutical care and microbiological testing from meeting demands, which can greatly increase the spread of drug-resistant organisms. Meanwhile, excessive workload may lead to CRPA worsening due to the job burnout and a lack of responsibility and sympathy among medical professionals [42].

### Implications and limitations

This study provided evidence on the association between increased number of medical professionals and reduced CRPA rate by focusing on the deep correlation and potential value between medical professionals and CRPA. Provincial-level panel data transcend the details of individual behaviours and help increase the understanding of the underlying connections [10].

Workload moderated the relationship between medical professionals and the CRPA rate, and increased medical professionals’ workload increased the CRPA rate. These results can provide evidence for the strategic and systematic allocation of different kinds of health professional resources in curbing CRPA.

Suggestions based on our findings are proposed: to increase the allocation of medical professionals for tackling CRPA, to relieve the overwork of medical professionals by guiding patients to visit hospitals rationally, and to reduce the CRPA rate for improving medical quality and safety.

There are also some limitations. First, the existing provincial balanced panel dataset contains data for only 4 years, and the data volume was relatively small, which may limit the robustness and statistical significance of the estimation; therefore, a dynamic panel model was not adopted. Second, since the characteristic variables of medical professionals and patients were not available, only the hospital level could be controlled in the model. Third, this study focused on the adequacy of medical professionals, but the economic and political characteristics of the medical professionals were not discussed. Increasing the number of medical professionals may improve CRPA performance but also means more input. If more data are available, economic and political factors should be considered to provide further evidence for medical professionals’ allocation and utilization. Fourth, we used the number of daily physician visits instead of other complex definitions as workload due to data unavailability, which may affect the generalization of the results.

## Conclusions

There was a significant association between an increase in the number of medical professionals and a decrease in the CRPA rate, and workload served as a significant moderator in this relationship. It was recommended that measures increasing medical professionals’ allocation and reducing their excessive workload should be implemented to curb AMR.

## Data Availability

The rates of CRPA we used in this study are publicly available on the China Antimicrobial Resistance Surveillance System (CARSS). Available at http://www.carss.cn/Report. The independent variables, moderating variable and control variable we used in this study are publicly available in China Health and Family Planning Statistical Yearbooks 2015–2018. National Health and Family Planning Commission. Statistics Yearbook of Health and Family Planning. Beijing: Peking Union Medical College Press,2015–2018.

## Abbreviations

AMR: Antimicrobial resistance
CRPA: Carbapenem-resistant *Pseudomonas aeruginosa*
coef.: Coefficient
*P. aeruginosa*: *Pseudomonas aeruginosa*
HAIs: Healthcare-associated infections
AMS: Antimicrobial stewardship
CARSS: China antimicrobial resistance surveillance system
PHY: The number of physicians per 1000 population
NUR: The number of registered nurses per 1000 population
PHA: The number of pharmacists per 1000 population
MCB: The number of clinical microbiologists per 1000 population
WORKL: The number of daily physician visits in hospitals
HOS: The number of tertiary hospitals
N: Number
SD: Standard deviation
Min: Minimum
Max: Maximum
STAFF: One type of medical professional, such as physicians, registered nurses, pharmacists, and clinical technicians
OLS: Pooled ordinary least squares
FE: Fixed effect
RE: Random effect
